# The War

**Published:** 1861-12

**Authors:** 


**THE WAR.**—The only sermon we have listened to, read, or heard of, since the war began, which expresses our two *leading* ideas on the subject, has been published, "by request," by W. S. Dorr, 101 Nassau street, New-York, and was preached on the day of the National Fast, Sept. 26th, 1861, at the Spring-street Church, by its pastor, Rev. Robert Davidson, D.D., who is at once an elegant writer, a cultivated scholar, and a Christian gentleman. If any one will take the pains to run over the headings of the fast-day sermons, so elaborately reported by the enterprise of the New-York dailies, it will be perceived that, "with one consent," they make the nation out a culprit, under the bastinado, for about every national crime under the sun. Our own minister prayed for "this wretched nation." As soon as we got home, we horrified our family by an out-and-out disclaimer of the whole thing, and told them we didn't believe a word of it, that the nation was "wretched," or a sinner above all others — that the Almighty was angry with it; on the contrary, it was nearer being a Christian nation than any other on the face of the earth, and enumerated its "works," the only solid sign of a true faith; its missionary, tract, and Bible operations; the millions expended voluntarily every year for objects wholly benevolent and religious; the very fact that a national fast was appointed and observed with great uniformity throughout the land showed that this was a government which looked through clouds, up to the Maker of the universe, to lead it aright. We contended that there was not only no evidence of the Divine displeasure, but the strongest that could be given of his smiles, in that the earth had never yielded a larger increase; never before was there such a call from abroad for every pound or bushel of produce our people could spare; and never, in our memory, had there been a year more free from epidemic diseases, than this same of eighteen hundred and sixty-one. The very cost of war was a blessing; for the rich, who had more than they could use, had to pay for it into the hands of the poor and industrious, and that thus would the land be flooded with money; and never since we have been a nation, has there been as much gold and silver in the country as at this hour. We said further, that the war was not to be regretted, and that God would bring a national good out of it, that would open up a more glorious future to it than could otherwise have been done. But fearing that we might be con sidered as getting up an opposition meeting to our honored and beloved minister, we broke short off, and began to talk about something else. Although, if we could have had our say out, we would have enunciated further, what Dr. Davidson, with great frankness, (and who, like the Doctor, could have lived for many years a near neighbor to the "Great Commoner," and not be frank and fearless too?) tells as a truth which multitudes only dare whisper, and which the magnates disclaim with particular pains, that in this war, "Slavery is the cause and object;" that the South intended thereby to extend its area, and to perpetuate it; and that the North intends, as the war has been forced on her, that Slavery shall not be extended, and that it shall cease on this continent. The Doctor argues that, if Slavery be a crime, and the North is seeking to cut it up by the roots, there is no reason why God should be angry with the North; but reason for the reverse; hence, with a wise and admirable sententiousness, he entitles his discourse: "A Nation's Discipline; or, Trials not Judgments."

Looking up, then, to God, as the Arbiter of nations; regarding him, as he truly is, more a merciful Father, toward both sides, than as a vindictive Judge, what are we to hope for? Simply that this war, to the whole people, is the entire "nation's discipline;" its "trial, not its judgment;" and we sincerely thank Dr. Davidson for embodying so grand an idea so tersely. And further, we are as certain of it as that the sun will rise to-morrow, that the United States, one and undivided, will come out of this war in such a way, that both North and South will feel at a future day, as the Editor has unwaveringly felt, since Sumter fell, that this war is the grandest event of the century—to be made the grandest by the overruling of that Merciful One who is the embodiment of "Love" to North and South also. What will be the exact manner of its solution, or how we would like it solved, would be but a mere opinion, but we have no fears of the result; on the contrary, we have had an abiding faith that the issue will be for God's glory and the whole nation's highest good. That there are evils connected with war, and that, as a nation, we have come short of our duty to Divinity, we unhesitatingly admit; but we cordially agree with Dr. D. in these two propositions, that as to North and South, this is a war of Slavery, and that it is a war of dis cipline and trial, not of judgment.

